# Wrist-ankle acupuncture for the treatment of acute orthopedic pain after surgery: a meta-analysis

**DOI:** 10.1186/s13018-023-03569-z

**Published:** 2023-02-15

**Authors:** Mengli Chen, Yiyin Xu, Xiuzhen Fu, Jiewei Xie, Xuewei Cao, Yisheng Xu

**Affiliations:** 1 Orthopedics Department of Knee, Guangdong Provincial Hospital of Traditional Chinese Medicine, 111 Dade Road, Guangzhou, 510120 China; 2 Trauma and Foot-Ankle Surgery, Guangdong Provincial Hospital of Traditional Chinese Medicine, 111 Dade Road, Guangzhou, 510120 China

**Keywords:** Wrist-ankle acupuncture, Acute pain, Orthopedic surgery, Meta-analysis, Randomized controlled trial

## Abstract

**Background:**

Wrist-ankle acupuncture (WAA) has been reported in the treatment of acute pain in orthopedic surgery. However, the effects of WAA on acute pain were controversial in the current studies. Therefore, the purpose of this meta-analysis was to critically evaluate the effects of WAA on acute pain in orthopedic surgery.

**Methods:**

Several digital databases were searched from the inception of databases to July 2021, including CNKI, VIP, Wanfang, CBM, Pubmed, Cochrane Central Register of Controlled Trials, Embase, Medline, and Web of Science Core Collection. The risk of bias was evaluated using the Cochrane collaboration criteria. The primary outcome indicators included pain score, pain killer dosage, analgesia satisfaction, and adverse reaction incidence. All analyses were performed with Review Manager 5.4.1.

**Result:**

A total of 10 studies with 725 patients with orthopedic surgery (intervention group: 361, control group: 364) were included in this meta-analysis. The results demonstrated that the pain score of the intervention group was lower than the control group, and the difference was statistically significant [MD = − 0.29, 95%CI (− 0.37, − 0.21), *P* < 0.0001]. Compared with the control group, the patient in the intervention group used smaller amounts of pain killer [MD = − 0.16, 95%CI (− 0.30, − 0.02), *P* = 0.02]. The satisfaction of patients on pain relief was also higher in the intervention group, and the difference was statistically [OR = 0.25, 95%CI (0.15,0.41), *P* < 0.0001].

**Conclusion:**

WAA has a certain effect on acute pain in orthopedic surgery, and the effect of WAA combined with other therapies is better than that of not using WAA therapy.

## Background

Over the past 20 years, the attention of professional societies to the importance of evaluation and treatment of acute pain has been a dramatic increased [1]. With sensory, cognitive, emotional, and social components, pain is an unpleasant experience [2]. Many daily activities can interfere with Pain, such as sleep, work, and social intercourse [3]. NSAIDs (non-steroidal anti-inflammatory drugs) are one of the most useful methods to control acute orthopedic pain after surgery is mainly controlled by. However, toxic and side effects can also be caused by drug therapy, like vomiting, constipation, urinary retention, dizziness, delirium, and other adverse reactions (4). Additionally, patients may also develop drug tolerance over time due to the long-term drug analgesia which will aggravate the economic burden for patients [5]. Therefore, more and more attention has been attracted to non-pharmacological therapies for acute orthopedic pain after surgery.

Invented by the Chinese, acupuncture is one of the complementary and alternative therapies. With a long history in China, acupuncture has been widely recognized and accepted [6]. Among them, In the 1970s, Zhang Xinshu, a professor at the second military medical university, invented a new kind of micro-acupuncture therapy named wrist-ankle acupuncture (WAA) [7]. By taking the needle entry points from the wrist or the ankle, WAA is a superficial acupuncture therapy that treats diseases through divides the body into six longitudinal zones. These zones correspond to six zones and needle entry points on the left and right wrists and ankles, respectively. Combined with modern neurology theory and traditional acupuncture theory, WAA is a new kind of electrical stimulation therapy [5]. Featured in simple operation and high safety, WAA can treat the symptoms all over the body, though the acupuncture site of WAA is limited to the wrist and ankle. According to the current clinical studies, WAA has been proven that it has significant efficacy in some clinical pain, like orthopedic pain, dysmenorrhea, soft tissue pain, and toothache [8]. According to Zhou’s research, WAA can achieve pain relief by increasing serotonin levels in the brain and raising the pain threshold [9]. What’s more, according to Chen’s study, the main mechanism of the analgesic effect of WAA may be associated with inhibiting the production of substance P and the promoting release of β-endorphins in plasma [4].

In recent years, studies comparison significantly increased the effect of WAA on acute orthopedic pain and drug therapy. Some studies showed that WAA or WAA plus drug therapy was more effective while others had opposite results. Therefore, the purpose of this meta-analysis was to critically assess the effect of WAA on acute orthopedic pain after surgery to provide a scientific reference for the development of an intervention strategy for acute orthopedic pain after surgery.

## Methods

According to cochrane handbook, a similar meta-analysis studied WAA [4] was used as a reference for this review.

### Searching strategies

This study systematically searched four digital databases which were China National Knowledge Infrastructure (CNKI), Wanfang, VIP, and China Biology Medicine (CBM). The keywords for searching are Wrist-Ankle Acupuncture "OR "Wrist Acupuncture" OR "Ankle Acupuncture" OR "wrist-ankle acupuncture"), pain OR ache OR soreness OR analgesia OR acesodyne, OR trauma OR sports injury OR postoperative. The search strategies for the databases are shown as follows.
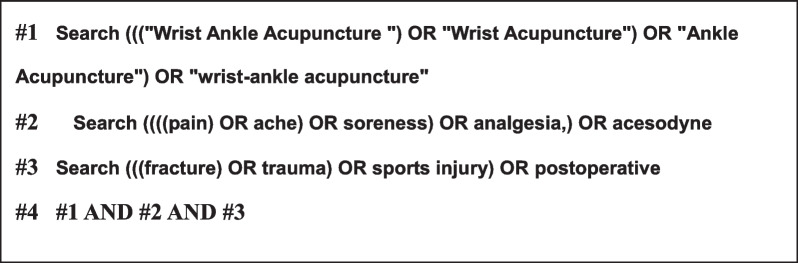


### Inclusion criteria

#### Participants

The participants included in the studies suffered from acute pain of postoperative, orthopedic pain, regardless of nation, region, or gender [10].

#### Interventions and controls

The interventions were wrist-ankle acupuncture alone or wrist-ankle acupuncture combined with other therapy. The control group was intervened with non-wrist-ankle acupuncture, placebo acupuncture, or body acupuncture.

#### Outcomes

The outcome indicators were pain score, the dosage of analgesics, satisfaction with pain relief, and adverse reaction.

#### Types of studies

Only random control trials were eligible.

### Exclusion criteria

The exclusion criteria were listed as follows: (1) Studies that are nonrandomized trials; (2) studies that did not use WAA as the major treatment; (3) studies that had repeated reporting with the same results; (4) studies had incomplete data; (5) studies didn’t set up pain measurements like pain scale or effective rate; (6) The procedure of wrist and ankle needle didn’t meet the national technical standards [11]; (7) the intervention periods were more than 30 days.

### Data extraction

Two researchers extracted and cross-checked the data independently. In case of differences, they would judge a third party. The basic data were extracted, including the first author of the literature, age of participants, number of participants, year of publication, intervention and control measures, indicators of effect evaluation, and incidence of adverse reaction. The author of the report would be contacted to obtain the information if the key information was missing.

### Risk of bias assessment

According to the Cochrane assessment tool, the risks of bias were evaluated by two reviewers independently. The assessment consists of the following seven domains “adequate sequence generation, blinding of outcome assessment, allocation concealment, blinding of participants and personnel, selective reporting, incomplete outcome data, and other bias. Each question can be rated as follows: yes (+), low risk of bias; unclear (?), unclear risk of bias; no (−), high risk of bias.

### Statistical analysis

Review manager 5.4.1 software was used for statistical analysis. Risk ratio (RR) was used for continuous data. Reporting and publication biases of the included studies were assessed by visually inspecting the asymmetry of the funnel plot. In each analysis, *I*^2^ was used to measure the statistical heterogeneity among the trials. If *P* > 0.05 and *I*^2^ < 50%, due to the homogeneity of the trials, the fixed effects model was used for analysis; We performed a meta-analysis to calculate risk ratios (RRs), absolute risk differences (ARDs), and 95% CIs using the Mantel–Haenszel statistical method. If zero events were reported for one group in comparison, a value of 0.5 was added to both groups for each such study. A random-effects model was used to pool the data, and statistical heterogeneity between summary data was evaluated using the *I*^2^ statistic. In addition, a subgroup analysis based on simple wrist-ankle acupuncture treatment and combined treatment was performed in this meta-analysis. Sensitivity analysis was performed as required. Publication bias was assessed by funnel plots and the Egger test for asymmetry when at least 10 trials were included.

## Results

### Study selection

**Fig. 1:**
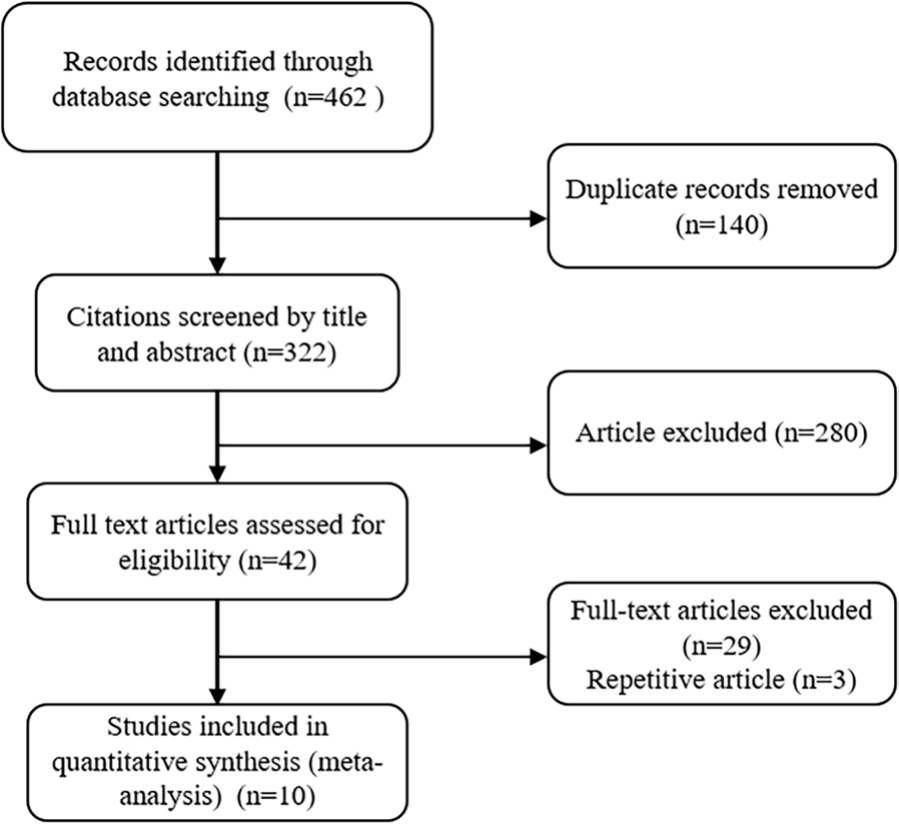
Flowchart diagram of trial identification and selection

An initial search of RCTs yielded 621 potential literature citations (Fig. 1). After the screening, 611 articles were excluded and 10 articles were examined in detail to assess their relevance.

### Overall study characteristics

Though 12 studies met the inclusion criteria, two studies could not be included because their relevant outcome indicators could not be included in quantitative research. All the studies were performed in china. The characteristics of the studies were shown in Table 1.

**Table 1:** Studies included in the systematic review

Author, year	Surgery	Sample (T/C)	The intervention for treatment group				Control group	Timing of pain measurement	Outcome indicator	Medical procedure	Adverse reactions
			Intervention model	Select acupoints for wrist and ankle needles	Timing of WAA	Retaining needle time					
Jiansong Chen [12]	UKA	70 (35/35)	WAA	Same side down 4	10 min before CPM	2 h	Routine analgesic nursing	1/3/7/14 days after surgery	VAS IL-6, TNF-α	Two each time, 14 days	None
Wenlong Li [18]	THA	68 (35/33)	WAA + PCA	Same side down 1, 4, 5	3 days before surgery	8 h	Painkiller	12 h/24 h/36 h/48 h/1/3/7/14 days after surgery	VAS Dosage of painkiller	One each time,10 days	13 Cases in experimental group and 28 cases in control group
Wenlong Li [17]	THA	73 (35/38)	WAA + low dosage of celecoxib	Same side down 1, 4, 5	3 days before surgery	8 h	Painkiller	4 h/8 h/12 h/1/2/3/4 days after surgery	VAS Dosage of painkiller	Once a day, This lasted until the 4th postoperative day	13 Cases in experimental group and 19 cases in control group
Jingjuan Zhu [21]	THA	68 (34/34)	WAA + Auricular point	same side down 1, 4, 5	1 days before surgery	8 h	Routine analgesic nursing	2 h/6 h/12 h/24 h/48 h/72 h//7/days after surgery	VAS Analgesia satisfaction	Once a day, 10 days	4 Cases in experimental group and 9 cases in control group
Jingjuan Tian [19]	THA	78 (39/39)	WAA + pain killer	Same side down 4, 5	After surgery	4–6 h	Painkiller	1 h/6 h/12 h/24 h/ after surgery术后1, 6, 12, 24 h	VAS Dosage of tramadol hydrochloride	Once a day	Not mentioned
Dongmiao [20]	Limb fracture	72 (36/36)	WAA	According to the fracture side	After admission to hospital	30 min	Painkiller	5 min before acupuncture, 10/15/30 min/1 h/24 h after acupuncture	Changhai pain measuring scale, dosage of tramadol hydrochloride, analgesia satisfaction	Once a day	1 Cases in experimental group and 4 cases in control group
Peiqian Lai [16]	Thoracolumbar internal fixation	60 (30/30)	WAA + PCA	Same side down 5, 6	1 day after surgery	30 min	Chinese and Western medicine pain nursing	12 h/24 h/36 h/48 h/3/4/5 days after surgery	VAS Dosage of analgesic pump, laboratory index	Once a day	3 Cases in experimental group and 11 cases in control group
Qiao Wang [13]	Calcaneus internal fixation	145 (48/48/59)	WAA	Same side down 1, 2	1 h after surgery, 6 h after surgery	1–24 h	Routine analgesic nursing	1 h/6 h/12 h/24 h after surgery	VAS	Once a day	Not mentioned
Min Zhang [15]	THA	84 (42/42)	WAA	Same side down 1, 4, 5	After surgery	2 h	Routine analgesic nursing	12 h/24 h/2/3/4/5 days after surgery	VAS	Once a day, 7 days	8 Cases in experimental group and 26 cases in control group
Hougang Xia [14]	Spine surgery	60 (30/30)	WAA	Same side down 5, 6	1 day after surgery	30 min	Routine analgesic nursing	12 h/24 h/36 h after surgery	VAS Dosage of analgesic pump; laboratory index	Once a day	8 Cases in experimental group and 16 cases in control group
Huihua Zhou [23]	TKA	74 (37/37)	WAA	Same side down 2, 3, 4	1 day after surgery, before CPM	4 h	Routine analgesic nursing	After admission to hospital and 1/3/5/7/14 days after surgery	NRS PSQI	Once a day, 7 days	2 Cases in experimental group and 17 cases in control group
Su [24]	Acute low back pain	60 (30/30)	WAA	Same side down 5, 6	After admission to hospital	30 min	Sham acupuncture	3 min before acupuncture, and 5/10/15 min during acupuncture, and 30 min after acupuncture	McGill, ETCS	Once a day	3 Cases in experimental group and 1 cases in control group

### Methodological quality and risk bias evaluation of the studies

**Fig. 2:**
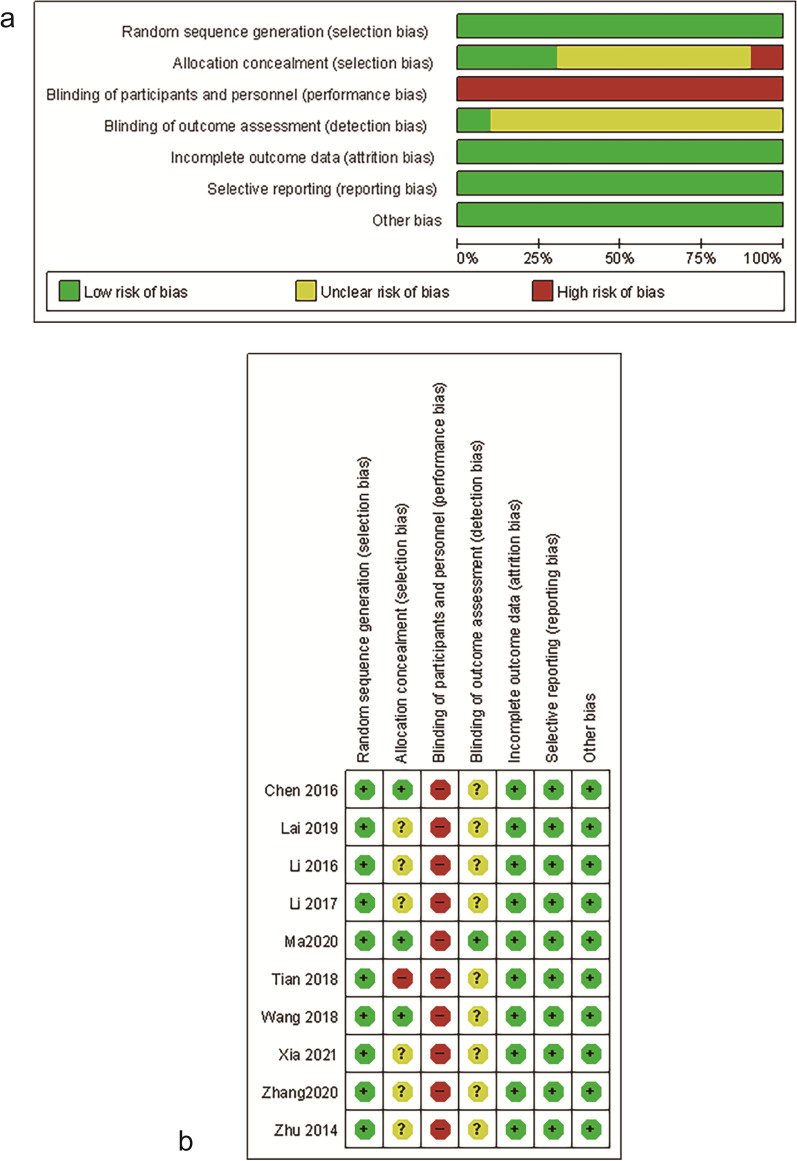
**a** Methodological quality evaluation of included studies. **b** Risk of bias assessment using the Cochrane tool

The methodological quality and risk bias assessment results of the included quantitative research are moderate. The random number table method was used in all the studies. Three studies used closed envelopes for random allocation. Since the control group used conventional analgesic nursing interventions or drug interventions and did not use fake acupuncture or body acupuncture treatment, the participants and the blindness of patients are not suitable for WAA intervention, so the risk of bias in all studies is high. Only one study reported blinded methods for evaluating results. The methodological quality evaluation of the included studies is shown in Fig. 2a, and the risk bias evaluation is shown in Fig. 2b.

### Results of the meta-analysis

#### Analgesic effect analysis base on VAS

##### Pure WAA group

**Fig. 3:**
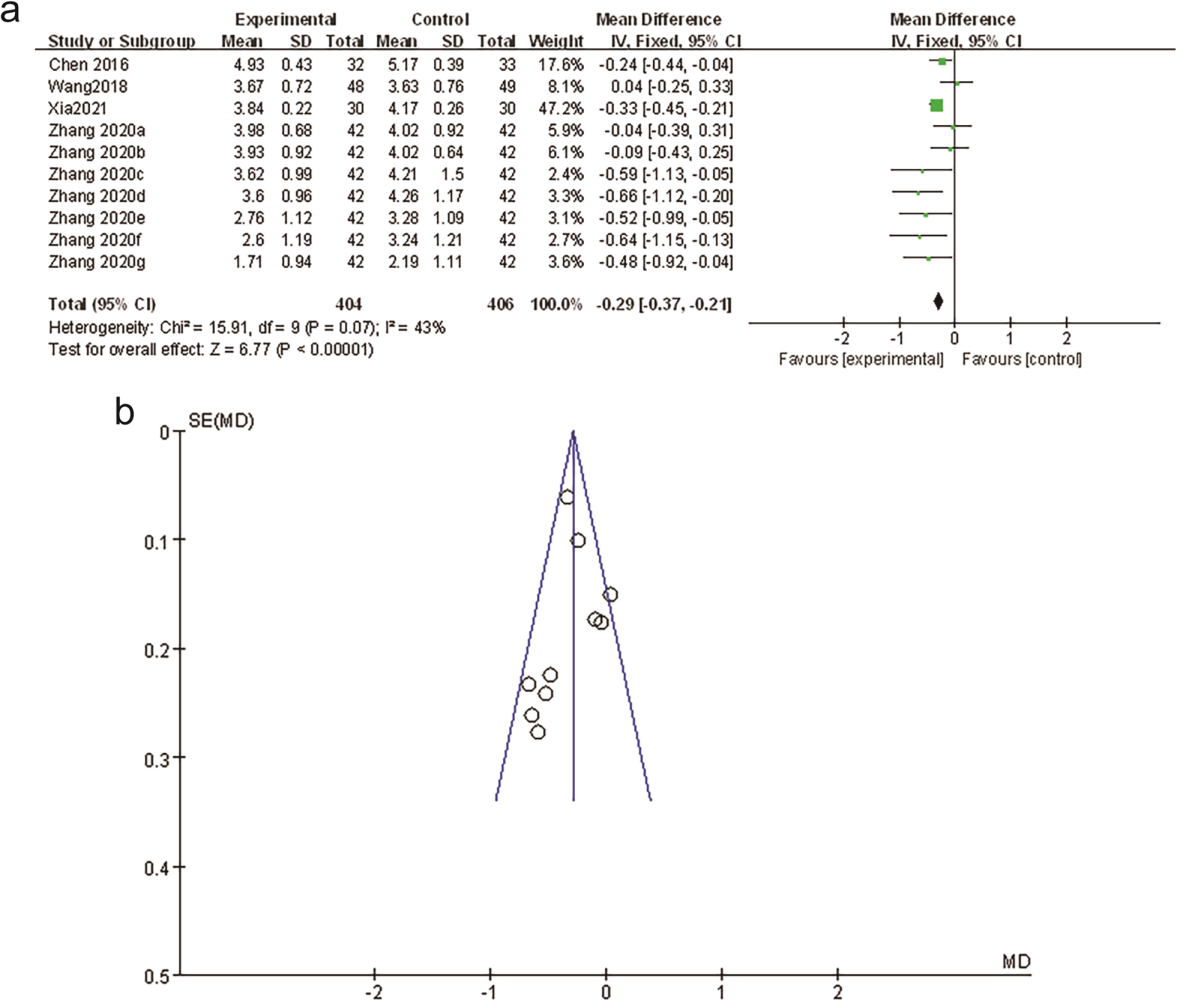
**a** Forest plots of WAA versus non-WAA therapy. **b** Funnel plots of pain relief rates: WAA versus non-WAA

Based on the VAS analgesic effects, four studies [12–15] reported that the pain score is lower in the ankle acupuncture group (Fig. 3a). The difference was statistically significant [MD = − 0.29, 95%CI (− 0.37, − 0.21), *P* < 0.0001]. The funnel chart (Fig. 3b) shows that the distribution of the literature included in the study's pain score comparison is still symmetrical, indicating that the bias is at an acceptable level and the analgesic effect of the wrist-ankle acupuncture group is better than that of the non-wrist-ankle acupuncture group.

##### Combined with other therapies

**Fig. 4:**
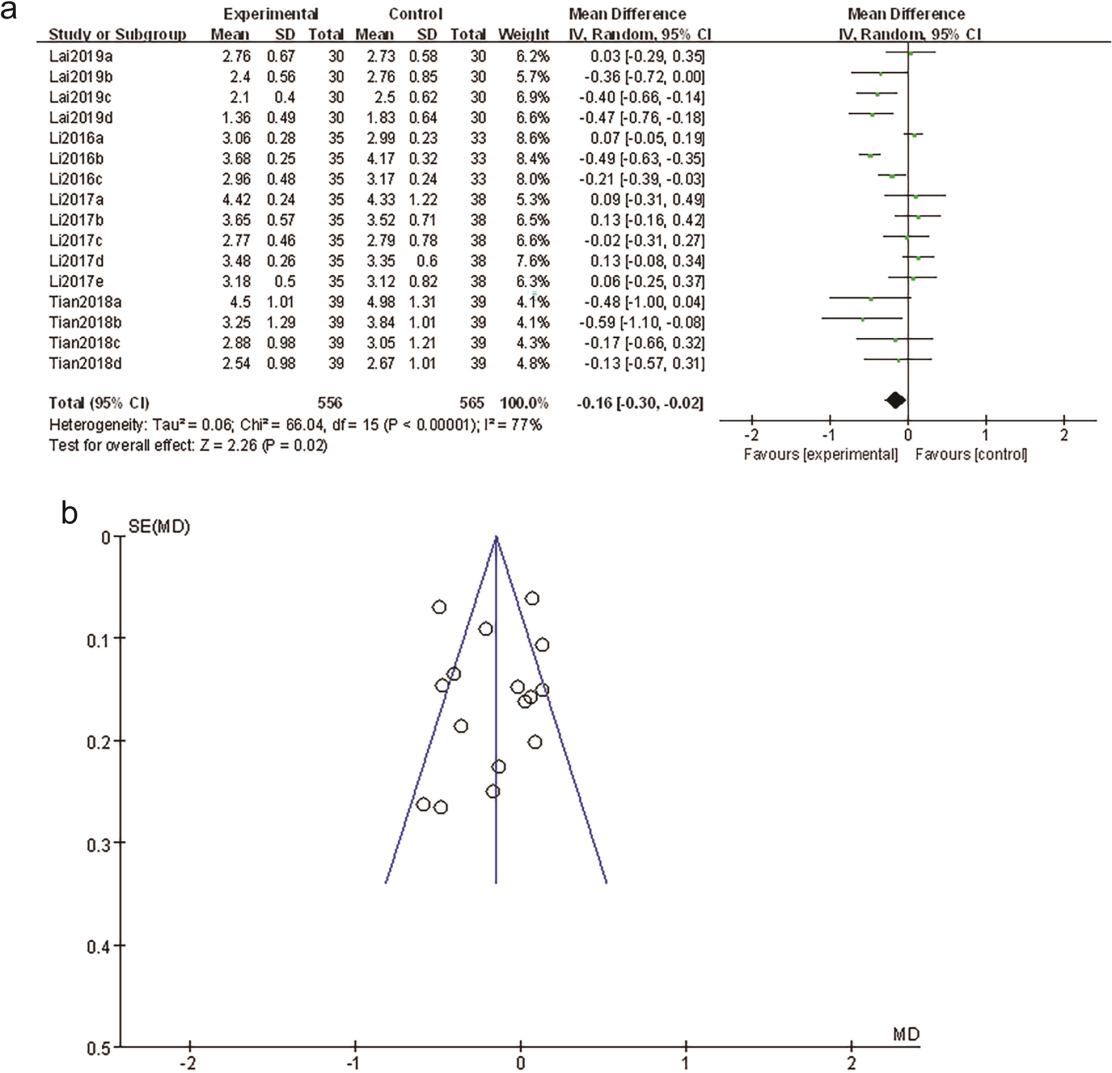
**a** Forest plots of WAA plus other therapy versus non-WAA therapy. **b** Funnel plots of pain relief rates: WAA plus other therapy versus non-WAA therapy

The analgesic effect was compared in four studies [16–19]. According to the results (Fig. 4a), the pain score in the WAA combined with other therapies group was lower than that of the non-WAA group, and the difference was statistically significant [MD = − 0.16, 95%CI (− 0.30, − 0.02), *P* = 0.02]. The funnel chart (Fig. 4b) shows that the distribution of the literature included in the study's pain score comparison is still symmetrical, indicating that the bias is at an acceptable level, and the analgesic effect of the wrist-ankle acupuncture combined with other therapies is better than that of the non-wrist-ankle acupuncture group.

##### Comparison of analgesic dosages

**Fig. 5:**
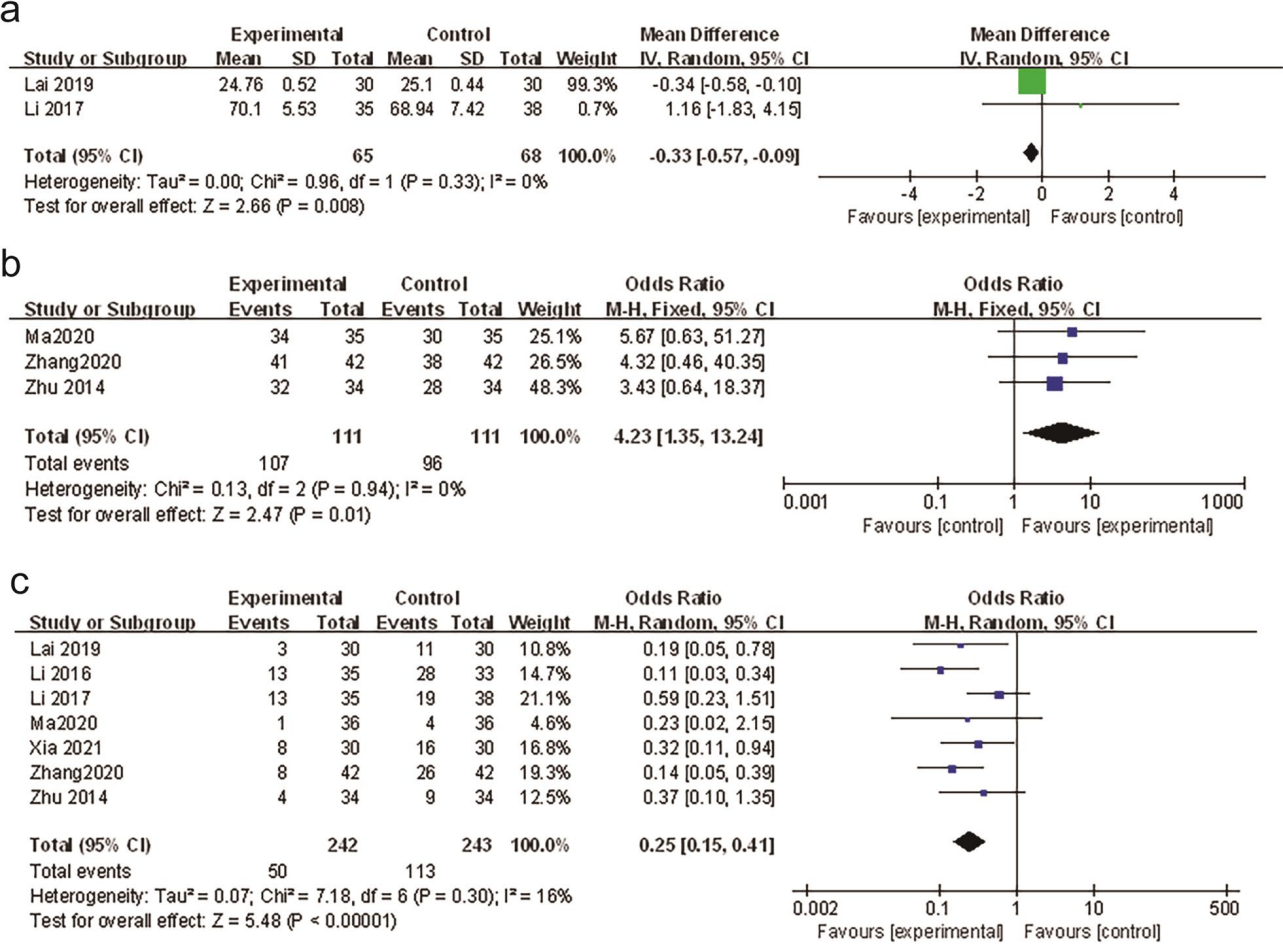
**a** Forest plots dosage of analgesic (WAA plus other therapy versus non-WAA therapy). **b** Forest plots of adverse reaction. **c** Forest plots of WAA analgesic satisfaction

Two studies [16, 17] compared the dosage of analgesic drugs, and 133 patients were included in the studies, including 65 in the experimental group and 68 in the control group. The results (Fig. 5a) showed that compared with the non-WAA group, the application of WAA can reduce the dosage of analgesic drugs, and the difference is statistically significant [MD = − 0.33, 95%CI (− 0.57, − 0.09), *P* = 0.008].

##### Analgesia satisfaction

Three studies [15, 20, 21] compared the satisfaction of analgesia effect after using WAA and 222 patients were included, 111 in the experimental group and 111 in the control group. The results (Fig. 5b) showed that the analgesic satisfaction of the WAA group was higher than that of the non-wrist-ankle acupuncture group, and the difference was statistically significant [OR = 4.23, 95%CI (1.35, 13.24), *P* = 0.01].

##### Adverse events reporting

Seven studies [14–18, 20, 21] reported adverse events. 485 patients were enrolled, including 242 in the experimental group and 243 in the control group. The adverse reaction rates were lower in the experimental group, and the difference was statistically significant (Fig. 5c) [OR = 0.25, 95%CI (0.15, 0.41), *P* < 0.0001]. The main adverse reactions in the WAA group were subcutaneous hemorrhage and fainting needles. The adverse reactions in the conventional analgesic care group were nausea and vomiting, urinary retention, dizziness, and drowsiness.

## Discussion

This meta-analysis included 10 studies conducted on a total of 725 participants (intervention group 361 patients, control group 364 patients). The random number table method was used in all studies. Three studies reported allocation concealment, and only one study reported blinding of outcome assessment. Five studies were pure WAA therapy and the other five studies were WAA combined with other therapies (combined treatment), such as combined with ear point pressure, the use of analgesic drugs, and the use of self-controlled analgesia pumps et al. The results showed that the analgesic effect in the WAA combined with other therapies group was better than that in the non-WAA group, and it also have a better analgesic satisfaction. Besides, in terms of adverse reactions, the experimental group was significantly lower than that of the control group, and the adverse reactions of the control group were mainly nausea and vomiting, urinary retention, vertigo, and, drowsiness, which was mainly related to the dosage of analgesic drugs in the routine analgesic care of the control group. In the WAA group, drug-related adverse reactions could be reduced by reducing the use of analgesics, and the main adverse reactions were subcutaneous bleeding and acupuncture fainting, which occurred in a small proportion. This study suggests that in the construction of a painless ward in the orthopedics department, it is beneficial to relieve the acute pain of patients by adopting WAA and combined with other traditional Chinese medicine therapies to control the symptom of the patients.

Despite our comprehensive review of the literature on the treatment of acute pain in orthopedic surgery with WAA, the present study still has some limitations. First, the quality of the studies included in this meta-analysis is mediocre, and the report on sequence generation and allocation concealment are incomplete. Most studies are considered to have a high or unclear risk of bias. Second, all studies were written in Chinese. Third, in the control group, conventional analgesic nursing, like sham acupuncture and body acupuncture was used, which made it difficult to achieve blindness for researchers and patients, thus affecting the accurate judgment of efficacy and easy to overestimate the efficacy.

## Conclusion

Results from this meta-analysis provide evidence that WAA or WAA combined with other therapies helps relieve acute pain in orthopedic surgery. Besides, WAA is a cheap and safe treatment, which is worthy of clinical promotion. But this meta-analysis included relevant and rigorous RCTs are insufficient; hence, higher quality and more rigorously designed clinical trials with large enough sample sizes are needed to further confirm our findings. Last, we were unable to conduct subgroup analysis, and there is a certain bias in the WAA combined with other therapies. This study has some implications for the future research direction of the application of wrist and ankle acupuncture in orthopedic pain.

## Data Availability

All data included in this study are available upon request by contact with the corresponding author.
